# The Late Dr. T. Romeyn Beck

**Published:** 1856-01

**Authors:** 


*The Late Dr. T. Romeyn Beck.*—Intimately connected with the later history of nearly every department of scientific literature, in this State, is the name beneath which we are writing. It is not our purpose, even did space permit, to follow the subject of this brief sketch through the many fields enriched by his labors, but simply to speak of his connection with the speciality to which this journal is more particularly devoted. Although his mind seems to have been directed to the subject of insanity upon the very threshold of his professional studies, it has received but a small share of his attention — sufficient, however, to have contributed largely to its literature and progress in this country.

Dr. Theodric Romeyn Beck was born at Schenectady, New York, August 11th, 1791. His grandfather, Rev. Derick Romeyn, a distinguished scholar of his day, was a professor of theology in the school of the Reformed Dutch Church, and one of the founders of Union College. By the death of Dr. Beck's father, his early care and education, and that of his four brothers, devolved upon their widowed mother. In the brilliant future and distinguished usefulness of her youthful charge we see the fruit of the piety, intelligence and energy of this truly excellent woman; and as the reward of all her care, we find her, in advancing years, the honored mother of one of the most talented families in the State.

Of these five sons, two died early—one a lawyer of great promise at St. Louis, and another, Nicholas F., who deceased while holding the office of Adjutant General under De Witt Clinton. Of the surviving brothers, Dr. John B. Beck, the distinguished author and physician, was for many years Professor of Materia Medica in the College of Physicians and Surgeons of New York, and died in that city in 1851. The remaining brother, Lewis C. Beck, was no less eminent, and at the time of his disease, two years since, was Professor of Chemistry in the Albany Medical College, and occupied the same chair in Rutger's College, New Jersey. To the general as well as professional reader the writings of both these brothers are well known, while the name of the latter is prominently associated with the preparation of the "Natural History of the State of New York," to which he contributed a valuable volume.

Dr. T. Romeyn Beck acquired the rudiments of his education in the Grammar School at Schenectady, under the more immediate supervision of his grandfather, and was graduated at Union College in 1807. Making choice of medicine as a profession, he soon afterwards commenced his studies with Drs. McClelland and Low, at Albany; but, induced by the superior advantages offered in the city of New York, he subsequently proceeded thither, and entered the office of Dr. David Hosack. He attended the lectures of the College of Physicians and Surgeons, then recently established, and received from that institution, in 1811, the degree of Doctor of Medicine, on which occasion he presented an inaugural thesis on the subject of Insanity.

This dissertation was immediately published, and received much merited attention. Although written at a time when but few in this country had devoted themselves particularly to the study of insanity, it exhibits, on the part of its author, a full appreciation of the importance of the subject, and a very intimate acquaintance with its literature. It is now out of print, the limited edition published soon finding its way into the hands of permanent possessors. The pamphlet contains thirty-four closely printed pages, and is inscribed to his uncle, Dr. John B. Romeyn, and Dr. David Hosack, and presented to his early preceptors, Drs. Low and McClelland, "as the first fruits of an education commenced under their care." After an introduction, with a brief detail of earlier investigations, and the various theories advanced by older writers to account for the phenomena of diseased mental action, follows a condensed history of the disease, its symptomatology, etiology, pathology, prognosis and treatment. In subsequent pages the medical jurisprudence of insanity is considered in reference both to the security of the public and the proper treatment of the patient.

This little volume, from the pen of "one whose opportunities of viewing the disease had been scanty, and whose information was derived principally from books," exhibits an intimate acquaintance with the literature of the subject, and the then only partially acknowledged wants of the insane, alike creditable to his character as a scholar and to his correct judgment.

Soon after his graduation he returned to the city of Albany, opened an office, and commenced the practice of his profession. His cultivated taste and studious habits soon brought him into intimate relation with scientific men of his day; and as early as 1813 we find his name upon the list of Counsellors of the "Society for the Promotion of Useful Arts," in connection with that of De Witt Clinton and others equally eminent. This association at that time held a high rank in the scientific world, and had enrolled upon its list of membership some of the most honored names in the State. It was a re-incorporation of the old "Society for the Promotion of Agriculture, Arts and Manufactures." Among his earlier and most successful efforts in this new and honorable field is the annual address, delivered by appointment before the society, at the Capitol, in the city of Albany, on the 3d of February, 1813. This production was more particularly directed to the public, the object being the more perfect development of the mineral resources of our country, or, as is stated in the preface, to exhibit at one view the mineral riches of the United States, with their various application to the arts, and to demonstrate the practicability of the increase of different manufactures whose materials are derived from this source. It was well calculated to awaken an increased interest in this important matter, and was received with great favor throughout the Union.

In 1815, Dr. Beck was appointed Professor of the Institutes of Medicine, and Lecturer on Medical Jurisprudence, in the College of Physicians and Surgeons of the Western District of New York, an institution then in the third year of its existence. The proximity of the College to the city of Albany enabled him to discharge his professional duties, and at the same time retain his medical practice, which he continued to do for some time.

Notwithstanding his many arduous duties, his interest in the progress of scientific investigation seems to have been unabated, and in the spring of 1819 he read before the Society for the Promotion of Useful Arts a most elaborate paper on Alum, which will be found printed with the transactions of the association. A short time previous he found his strength unequal to the laborious duties of his profession; and, on account of his apprehension of ill-health, and, perhaps, in indulgence of his increasing taste for literary pursuits, he abandoned the general practice of medicine entirely, and, in 1817, was appointed Principal of the Albany Academy, an institution collegiate in its character, and occupying a high literary standing. Teaching was especially adapted to his taste; and, under his enlightened management, for more than a quarter of a century, the academy unvaryingly maintained a most elevated rank among similar institutions. Notwithstanding his connection as Principal with the Albany Academy, he seems to have retained his Professorship at the College of Physicians and Surgeons, and, in 1824, delivered an introductory lecture "On the Utility of County Medical Institutions."

In 1829 Dr. Beck was elected President of the Medical Society of the State of New York, and, at the meeting of the Society, at Albany, delivered the annual address, on the subject of "Medical Evidence." Continuing in office several years, he pronounced, on similar occasions subsequently, two addresses—one upon "Medical Improvements," and the other upon "Small-pox," all of which will be found in the volume of "Transactions" for the respective years.

Since 1841 he has filled the honorable situation of Secretary to the Board of Regents of the University of New York; and, besides the multiplied duties connected with that position, has had devolving upon him, as *ex officio* Secretary to the Trustees of the State Library, a large share of its management. The complete and well arranged catalogue of the Library, and the interesting and comprehensive reports of the Board of Regents, bear the impress of his untiring application and devotion to the important interests over which that distinguished body presides.

Dr. Beck has always been a man of great and enlightened public spirit, ever ready to countenance and promote whatever tended to secure the highest interest of the community. This spirit and his natural benevolence have enlisted him ardently in the great public charities, either in their establishment and organization, or in the subsequent management of their affairs, His "Statistics of the Deaf and Dumb," read before the Medical Society of the State of New York, was the fruit of this philanthropy, and was most powerful in directing the attention of the public to the wants of this afflicted portion of the community.

Dr. Beck was appointed one of the Managers of the New York State Lunatic Asylum, by the act of its organization, in April, 1842, and has been reappointed by the Governor and Senate, at the expiration of each successive tri-annual period, until the present time. Upon the death of Mr. Munson, in the spring of 1854, he (although a non-resident member) was unanimously elected President of the Board. The institution has, at all times, had the advantage of his wise counsels, efficient aid, and ardent devotion, and of his presence and immediate co-operation with his associates whenever demanded by matters of unusual or special importance. Here, as well as in all other similar positions, he has ever consulted the highest and most enduring good of the interests committed to his charge, without regard to the prejudices or the more apparent benefits of the hour or the day, or any mere personal claims or advantages. His wisdom and experience, his independence, decision and energy, and his unflinching integrity, have made him a most valuable guardian of all the affairs of this great public charity.

It is, however, with Dr. Beck as a writer, that we have at present especially to do, and we will close this sketch by a notice of his editorial connection with this Journal, and his great work on Medical Jurisprudence.

In April, 1844, the first number of the American Journal of Insanity was issued from the press, occupying an entirely new field in the medical literature of this country. The generous motive which led Dr. Brigham, its founder and first editor, to assume, in addition to his onerous duties as Superintendent of a large asylum, the labor and responsibility of its establishment, is well known to most of our readers. To many of his colleagues and professional friends he was largely indebted for encouragement in his undertaking, and for much valued and gratefully acknowledged assistance; among them, Dr. Beck, who, deeply interested in the attainment of the ends at which the Journal aimed, warmly seconded his efforts, and, among other engagements, found sufficient time to contribute frequently and ably to its pages. After Dr. Brigham's death, the Managers of the State Lunatic Asylum, aware of the importance, to any speciality, of a periodical devoted to its advancement and interest, assumed the entire responsibility of its publication, and, by their unanimous request, induced Dr. Beck to edit the ensuing volume. He gave his consent, hoping at the close of the year to be relieved of a care which, with his other numerous duties, was a heavy tax; but, in the absence of any other arrangement, he continued to conduct it until the close of the last volume, when "advancing years and more imperative duties" compelled him to relinquish his editorial connection.

In the theme of his inaugural dissertation at the Medical College, and in the subject of many of his literary efforts, we perceive how early and closely his attention has been drawn to insanity and its legal relations. From a knowledge of his character, it is very natural to suppose that this interest was awakened, not only to the intrinsic merit of the subject, but also by the then very general feeling that this department of medical literature was indeed most barren. How well he succeeded in his efforts to supply this deficiency is evidenced by the multiplied editions of his "Medical Jurisprudence" which have already been called for. Since its first issue from the press, in 1823, in two large octavo volumes, of nearly two thousand pages, it has passed through five American, one German, and four London editions. The favorable reception of this work in foreign countries, at a time when national feeling in the medical world was stronger than at any previous or subsequent period, shows how completely its merits disarmed every prejudice. Says a bibliographer, in a notice of the German edition: "Among the numerous and unequivocal evidences of the very high estimation in which Dr. Beck's 'Elements of Jurisprudence' are held by the profession in Europe, their translation into the German language must be regarded as the most flattering and decisive indication of their true value. In no country has this interesting and varied science been prosecuted with such unabated zeal, or have so much research and learning been elicited on its several topics, as in Germany. From the time of Zacchias, indeed, to the present day, it has been the favorite object of study with German physicians, and their opinions of the merit of any treatise on the subject are therefore entitled to the highest weight and the most respectful consideration. Proud are we, therefore, to see them prize the performance of our learned countryman so highly as to deem it worthy of transfusion into their vernacular tongue. In his native language his work is without a parallel."

His labors in this field did not cease with the publication of his great work, but, for many years afterwards, besides the emendation and supervision of subsequent editions, he contributed largely upon the same subject to various medical periodicals. A distinguished writer, in reviewing a copy of the tenth edition, for Hay's American Journal of Medical Science, remarks: "The pages of this Journal, for years past, have borne constant evidence of the untiring and *invaluable* research of Dr. Beck, whose observations and extracts from foreign and domestic sources have filled that portion of it devoted to medical jurisprudence; and the writer of the present notice bears his testimony to the same effect; for, having taken much interest in the subject, and consequently had occasion to examine the journals, he found it impossible to furnish a single novelty in this department in which he had not been anticipated by Dr. Beck." In both the medical and legal periodicals of the day there have, from time to time, with successive editions of his work, appeared many and varied notices and reviews—flattering evidence of its merit, and the high estimation of both professions. From some of these it would give us pleasure to extract; but the work has already received the stamp of worth, has taken its place as a high authority, and acquired for itself and its author a most extended reputation.—*American Journal of Insanity.*

